# Prosodic hierarchy and the encoding of modality: a study of L2 French statements and commands by Mandarin learners

**DOI:** 10.3389/fpsyg.2026.1798654

**Published:** 2026-04-14

**Authors:** Xintong Chu, Pierre Larrivée

**Affiliations:** 1 School of Western Languages, Mudanjiang Normal University, Mudanjiang, China; 2 Department of Language Sciences, University of Caen Normandy, Caen, France

**Keywords:** French prosody, Mandarin learners, Modality-Prosody Acquisition Hierarchy, prosodic merging, tone-intonation Interface

## Abstract

The interface between lexical tone and phrase-level intonation constitutes a complex functional interface constraint in Second Language Acquisition (SLA). This study investigates the acquisition of French prosody by Mandarin learners (*n* = 35) at two proficiency levels compared to a native baseline (*n* = 8), identifying a developmental dissociation between structural and pragmatic functions. Utilizing Linear Mixed-Effects Models (LMM), we analyzed four acoustic parameters—Global Pitch Span, Final Pitch Span, Final Expansion, and Final Lengthening—across a factorial design crossing modality (Statement vs. Command) and utterance length. The results reveal a bifurcated acquisition trajectory: while intermediate learners demonstrate convergence toward native-like macro-prosodic framing (successfully expanding global pitch envelopes for structural demarcation), they reach a persistent plateau in micro-prosodic pragmatic encoding. Specifically, learners failed to implement the sharp nuclear reshaping and pitch intensification required to signal directive force, resulting in a systematic “prosodic merging” of modalities. Based on these findings, we propose the Modality-Prosody Acquisition Hierarchy (MPAH), which posits that the automation of a stable structural frame (Tier 2) serves as a functional prerequisite for the high-resolution modulation of illocutionary intent (Tier 3). These findings refine the Prosodic Transfer Hypothesis by isolating the selective processing constraints at the tone-to-intonation transition and offer a tiered roadmap for pedagogical intervention.

## Introduction

The acquisition of prosody represents a profound typological challenge when the learner’s first language (L1) and the target language (L2) employ fundamentally different systems for encoding meaning. A primary example of this divide is the contrast between intonation languages like French and lexical tone languages like Mandarin Chinese. In French, pitch variations are primarily phrasal phenomena, mobilized to signal communicative modalities ranging from neutral assertions to directive commands (Di Cristo, 2016; Mertens, 2019). Conversely, Mandarin’s prosodic space is constrained by its lexical tone system, where pitch variations are localized at the syllable level to distinguish word meanings (Chao, 1968; Lin, 2006).

This functional mismatch creates a critical interface constraint in phonological development. While previous research has extensively mapped segmental errors, the acquisition of phrasal-level modality markers remains a complex and persistent developmental challenge. In French, the distinction between a statement and a command often relies exclusively on prosodic cues rather than morphological markers, placing a high functional load on intonation (Martin, 2018). For Mandarin learners, this requires a fundamental re-ranking of cues: they must learn to suppress syllable-level tonal variations in favor of the long-range pitch trajectories required by French phrasal intonation. This study argues that the functional competition between L1 lexical tone and L2 intonational resources (Liang and van Heuven, 2009) makes the acquisition of French modality a critical test case for understanding the linguistic and cognitive constraints on L2 prosody.

To understand this acquisition process, it is essential to situate the analysis within the Prosodic Hierarchy Theory (Selkirk, 1984; Nespor and Vogel, 1986). In French, a language with no lexical stress, prosodic prominence is organized around the Accentual Phrase (AP). The realization of sentence modality—specifically the distinction between declarative statements and directive commands—relies heavily on the manipulation of AP boundaries. Typically, declaratives are signaled by a gradual declination ending in a low-falling boundary tone (L%), whereas commands require a more abrupt and sharp falling contour realized on the nuclear syllable of the final AP, often accompanied by increased subglottal pressure and rhythmic intensity (Di Cristo, 2016; Martin, 2018), and so that theory allows us to systematically isolate the specific hierarchical level—namely, the phrasal boundary—where the functional competition between Mandarin syllable-bound tones and French macro-prosodic markers occurs.

## Theoretical background and state of the art

The intonation patterns of statements and commands are categorized as conclusive contours, which are associated with the modality of the sentence—that is, the speaker’s communicative attitude toward the utterance, often intended to elicit a reaction from the listener. From this perspective, statements correspond to the declarative modality, whereby the speaker presents information or asserts a proposition. Commands, on the other hand, are connected to the notion of “insistence,” which may relate either to the semantic content of the sentence or to the pragmatic context of the speech act, which suggests an action expected on the part of the hearer (König and Siemund, 2007). In this sense, the command is often viewed as an insistent variant of the declarative (Martin, 2018).

The intonation of statements is often considered the default model, whereas the intonation of commands is typically derived from the imperative that prototypically expresses such commands—the imperative form being traditionally listed in verb conjugation tables, although its structures are often borrowed from the indicative. Previous studies on the intonation of French statements unanimously agree that, in utterances with broad focus, the final contour generally displays a slight downward movement. Delattre (1966) described this as a descent from level 2 to level 1 (according to his five-level musical scale), a pattern that has been confirmed by subsequent research (Rossi, 1999; Di Cristo, 2016; Martin, 2018; Mertens, 2019). This downward movement creates a declination effect, or in some cases, a supra-declination effect characterized by final lowering (Post, 2000; Di Cristo, 2016). A low flat contour may also occur (Jun and Fougeron, 2000; Delais-Roussarie et al., 2015).

Regarding the intonation of imperatives, Delattre (1966) observed that imperatives typically end with a falling intonation, descending from level 4 to level 1. Di Cristo (2016, p. 420–421) further noted that this gradual fall often characterizes the overall melodic contour of the sentence, with the final syllable exhibiting either a minor fall or a flat contour, depending on the nature of the speech act. For instance, polite invitations frequently employ a flat contour (Di Cristo, 2016). Another commonly observed melodic pattern for French imperative intonation involves an intonational rise on the pretonic syllable (Hi), followed by a final drop. According to Delais-Roussarie et al. (2015) and Di Cristo (2016), imperatives can also exhibit a rise and fall realized on the tonic syllable—typically described as “(L) H* L%.” This pattern is generally associated with commands that carry weaker directive force, such as polite requests or suggestions.

The acquisition challenge for Chinese-speaking learners is rooted in a fundamental typological mismatch between the L1 and L2 rhythmic systems. Mandarin Chinese is traditionally classified as a syllable-timed (or syllable-counting) language, where each syllable is associated with a specific lexical tone that constrains its fundamental frequency (F0) trajectory (Chao, 1968; Lin, 2006). In contrast, while traditionally classified alongside syllable-timed languages in early typologies, contemporary phonological frameworks increasingly characterize French prosody as being fundamentally phrase-based or exhibiting phrase-level timing (Jun and Fougeron, 2000; Michelas and D’Imperio, 2010; Delais-Roussarie et al., 2015; Santiago, 2019). In this system, rhythmic duration and pitch movement are distributed across larger syntactic-prosodic units (such as the AP), with stress robustly anchored to the right boundary rather than being uniformly tethered to individual syllables.

Within the hierarchy of prosodic units, Mandarin learners often exhibit a syllable-independent processing bias. Because the L1 system requires the preservation of tonal identity for every lexical item, learners may struggle to inhibit their L1 tendency to anchor pitch to individual syllables, making it difficult to execute the cohesive, utterance-level contours characteristic of French. This conflict becomes particularly acute in the realization of commands. While French native speakers utilize a top-down approach, planning a cohesive melodic contour that spans the entire utterance to signal directive force, Mandarin learners may approach the sentence bottom-up, treating each word as an isolated tonal target.

Research on the prosody of declarative sentences produced by L2 French learners has primarily focused on the prosodic deviations observed in Korean and Norwegian learners. Norwegian learners of French as an L3 frequently produced rising contours or truncated descending realizations (L%) (Steien and Lyche, 2016). Grandon and Yoo (2014) analyzed prosodic errors across two parameters: intonation and rhythm. Their findings showed that Korean learners struggled to appropriately elongate the final vowel of the AP in SVO declarative utterances. To compensate, they often merged the verb phrase with the object complement, leading to re-segmentation, an increased number of APs, and ultimately a disruption of prosodic structure, which negatively impacted fluency. Although their F0 contours were sometimes close to native-like realizations, errors were concentrated around AP boundaries, particularly between verbal phrases and objects. This persistent influence of L1 structural bias is further corroborated by recent investigations into L2 processing strategies among diverse typological pairs (Meng and Wang, 2009; Song and Chang, 2026); for instance, Song and Chang (2026) found that L1-Korean learners of Chinese struggle with the structural mapping of double-topic sentences due to entrenched L1 processing habits. These findings mirror our observation that Mandarin learners’ bottom-up processing—driven by L1 tonal requirements—prevents cohesive, top-down phrasal planning in French.

The French imperative modality is not merely a semantic category but a prosodic one, requiring distinct acoustic prominence at the AP-final boundary. Previous research demonstrates that native speakers employ a combination of increased intensity, terminal pitch falls, and precise temporal markers to encode insistence (Martin, 2018). However, for Mandarin-speaking learners, the L1 syllable-timed processing bias—often characterized in the literature as a perceptual filter (e.g., the Stress Deafness Hypothesis, Dupoux et al., 1997)—appears to constrain both the perception and production of phrase-final lengthening and global declination required for target-like imperative contours. Rather than producing a coherent descending slope, learners frequently exhibit a series of non-integrated pitch peaks, leading to systematic prosodic oversegmentation. This lack of phrasal integration may result in a rhythmic profile that obscures the directive intent of the utterance, potentially leading listeners to interpret the command as a sequence of hesitant statements. By comparing the acoustic realizations of statements and commands, this study aims to delineate how the interference between Mandarin’s syllable-based tonal constraints and French’s phrasal-level intonation shapes the developmental trajectory of L2 prosodic competence.

The acquisition of L2 intonation involves more than phonetic imitation; it requires a systematic re-ranking of linguistic cues. Within the framework of Cue Weighting (Ellis, 2006), learners must discern which acoustic parameters—pitch, duration, or intensity—carry the highest functional load in the target language. For Mandarin learners of French, this is complicated by a divergent functional distribution of pitch. In Mandarin, pitch serves a lexical function, with its functional load concentrated at the syllable level (Chao, 1968). In French, conversely, pitch is primarily post-lexical, functioning to encode syntactic structure and sentence modality (Jun and Fougeron, 2000; Di Cristo, 2016). This functional reallocation poses a significant mapping challenge. When Mandarin learners produce French, lexical-tonal interference often occurs, whereby F0 remains anchored to individual syllables rather than being mobilized for the long-range, phrasal-level trajectories required by the target system. The contrast between statements and imperatives thus serves as an ideal test case for analyzing this functional transition.

The imperative modality in French represents a unique area of investigation because of its syntactic transparency and high reliance on prosodic cues. Unlike many other languages that employ specific mood particles or mandatory morphological marking, the French imperative often relies on the deletion of the subject pronoun [e.g., *Tu parles*. (Statement) vs. *Parle*! (Command)]. In many phonetic contexts, the verb form itself remains ambiguous between the indicative and the imperative (e.g., *Tu chantes*. vs. *Chante!* are phonetically identical /ʃãt/). Consequently, prosody becomes the sole linguistic vehicle for distinguishing a neutral proposition from a directive inquiry or command. The illocutionary force of a command—its power to prompt action—is encoded in specific prosodic markers: a sharp falling contour (L%) (Delais-Roussarie et al., 2015), often accompanied by increased subglottal pressure and distinctive rhythmic timing on the nuclear syllable (Martin, 2018). For the L2 learner, the functional load of prosody in commands is therefore expensive in terms of cognitive resources. While statements can be produced using a default, low-effort declination, commands require an intentional, top-down execution of prosodic prominence to ensure communicative success. If a learner fails to re-weight prosodic cues and instead reverts to a flat or rising contour (perhaps due to L1 pragmatic interference or syllable-timed habits), the communicative intent undergoes a total breakdown, as the listener may perceive the command as a hesitant statement or a truncated question. By analyzing the developmental trajectory of these two modalities, this study explores how Mandarin learners navigate the trade-off between their L1-based lexical-pitch focus and the L2-required phrasal-prosodic focus.

To navigate this functional transition, we integrate the aforementioned Prosodic Hierarchy with the L2 Intonation Learning Theory (Mennen, 2015). According to this combined framework, acquiring French prosody is not a uniform process but a stratified one. Theoretically, the establishment of macro-prosodic structural boundaries (e.g., the Intonational Phrase envelope) must serve as a prerequisite before learners can effectively manipulate micro-prosodic features (nuclear reshaping) for pragmatic intent. Furthermore, because executing fine-grained pragmatic pitch variations in an L2 requires substantial processing resources, we predict that increased syntactic length will naturally force a trade-off, compelling learners to abandon complex pragmatic markers in favor of maintaining basic structural continuity.

Based on the theoretical framework discussed above, the present study tests the following four hypotheses:

*H1*: The Prosodic Merging Hypothesis

We hypothesize that Chinese-speaking learners will exhibit a phenomenon of prosodic merging, failing to significantly differentiate commands from statements through global and local pitch modulation due to L1 tonal constraints.

*H2*: The Hierarchical Fragmentation Hypothesis.

Regarding rhythmic organization, learners will exhibit hierarchical fragmentation (oversegmentation of APs), which will decrease as proficiency increases and phrase-based planning is automated.

*H3*: The Acoustic Imbalance Hypothesis.

Following the structural dependency outlined in the Prosodic Hierarchy, we predict an asynchronous acquisition trajectory. Specifically, learners will master macro-level “prosody for structure” (global pitch envelopes for demarcation) prior to mastering micro-level “prosody for pragmatics” (sharp nuclear reshaping for illocutionary force).

*H4*: The Complexity-Modality Trade-off.

Because mapping novel pragmatic functions onto L2 prosody necessitates significant computational resources, we predict that increased syntactic complexity (utterance length) will induce a functional processing constraint. Consequently, the prosodic gap between native and learner productions will be most pronounced in the long-command condition; specifically, learners will prioritize the maintenance of structural stability at the expense of local pragmatic precision, leading to a systematic trade-off between phrasal prosodic integration and illocutionary force.

## Methods

To empirically test these hypotheses, we designed a controlled production experiment. The following section outlines the participants, stimuli, and the multi-dimensional acoustic analysis implemented.

### Participants

A total of 43 participants were selected and divided into three groups: native French speakers (*n* = 8), beginner L2 French learners (*n* = 16), and intermediate L2 French learners (*n* = 19). The native speaker group comprised five Master’s students in Language Sciences from the University of Caen and three French as a Foreign Language (Français Langue Étrangère, FLE) instructors from the Le Mans University, with a mean age of 27.38 years. They were all native speakers of Hexagonal French and reported no speech or hearing impairments. The two learner groups consisted of native Mandarin speakers from Beijing, Tianjin, Hebei, and Shandong, China, ensuring a homogeneous tonal background. They were undergraduate students majoring in French at Hebei University of Technology. Proficiency levels were operationalized based on cumulative instructional hours and institutional placement aligned with the Common European Framework of Reference for Languages (CEFR): Beginners (Group A): First-year students enrolled in CEFR A1–A2 level courses, with approximately 150–200 h of formal French instruction; Intermediate learners (Group B): Second-year students enrolled in CEFR B1–B2 level courses, with approximately 600–800 h of formal instruction. This categorical operationalization of proficiency, rather than a continuous metric, was deliberately chosen to capture distinct developmental stages (A-level vs. B-level milestones) rather than assuming a strictly linear trajectory of prosodic acquisition. To ensure sample representativeness, participants were selected based on their performance in the most recent departmental final examination (top 40% percentile) and instructor recommendations. None of the learners reported prior immersion experience in a French-speaking country. The mean ages for Group A and Group B were 18.81 and 20.37 years, respectively.

Given the inclusion of three FLE instructors in the native group, a preliminary analysis was conducted to rule out the influence of teacher talk (e.g., hyper-articulation or reduced speech rate). Independent samples t-tests compared the instructor subgroup (*n* = 3) against the student subgroup (*n* = 5) across the four dependent variables. No significant differences were found for any acoustic parameter (all *p* > 0.05), nor for Speech Rate. This lack of variation suggests that the scenario-priming protocol successfully elicited naturalistic, communicative speech rather than didactic vocalizations.

Consequently, the eight native speakers were treated as a single, homogeneous baseline group. While a sample size of *n* = 8 may appear relatively small for broad sociolinguistic generalizations, it is widely considered robust and standard practice for highly controlled, laboratory-based acoustic phonetic production studies (e.g., Jun and Fougeron, 2000; Michelas and D’Imperio, 2010). Furthermore, the high density of observations per participant—yielding a rich dataset of acoustic trajectories across multiple controlled conditions—provides sufficient statistical power to establish a normative baseline. This adequacy is empirically supported by our data (detailed in section 3.4), which demonstrates that the native cohort occupies a tightly clustered, high-precision phonetic space with lower inter-speaker variability compared to the learner groups, thereby establishing a statistically stable normative baseline for our inter-group comparisons.

### Stimuli

The corpus consisted of 16 target sentences organized in a 2 × 2 factorial design, crossing Sentence Modality (Statement vs. Command) and Utterance Length (Short vs. Long). The full list of stimuli and their corresponding pragmatic contexts is provided in Appendix.

The stimuli were curated based on three critical criteria to ensure both phonetic precision and pragmatic validity:

1. Structural and Metric Specifications: Utterances were categorized by syllabic length to impose varying degrees of planning load. The Short condition comprised utterances ranging from 3 to 5 syllables (e.g., *Il pleut fort*.), simulating rapid, high-frequency communicative acts. The Long condition consisted of utterances ranging from 8 to 12 syllables (e.g., *Lavez-vous les mains avant de manger*.), requiring more complex, long-range prosodic integration across multiple rhythmic groups.
2. Phonological Constraints: To ensure the accuracy of pitch extraction, the stimuli were constructed with a high density of sonorant and voiced segments (vowels, nasals, liquids). This phonological control minimizes micro-prosodic perturbations (e.g., F0 dips caused by voiceless obstruents) and prevents tracking errors in the pitch contour analysis.
3. Syntactic and Pragmatic Design: To maximize ecological validity, the stimuli employed naturalistic syntactic structures appropriate for each modality. Statements followed standard declarative syntax (SVO), while Commands utilized the imperative mood (characterized by verb-initial structures or subject deletion). Although syntax contributes to modality marking, the specific sentences were selected because their illocutionary force—the speaker’s intent to compel action—relies heavily on prosodic reinforcement (e.g., nuclear pitch reshaping) rather than morphosyntactic particles alone. To ensure that participants produced these with the intended directive force, each target sentence was paired with a specific situational prompt (see Procedure).

For the purpose of terminological clarity, Command is used throughout this study to denote the directive modality (contrasted with Statement), while Imperative refers specifically to the grammatical structures and mood employed to realize these commands.

### Procedure

Data collection took place in quiet, sound-attenuated rooms at the University of Caen (France) and Hebei University of Technology (China). Recordings were digitized directly onto a laptop computer at a sampling rate of 44.1 kHz (16-bit resolution) using Praat software (Boersma and Weenink, 2024). To minimize amplitude variance and ensure a constant mouth-to-microphone distance, participants wore a high-quality headset microphone (Logitech H390) adjusted approximately 2–3 cm from the corner of the mouth.

The experimental session consisted of three phases:

1. Familiarization: Participants first reviewed the list of target sentences and situational prompts silently. They were encouraged to consult the *Dictionnaire du français contemporain illustré* (2012) for any unfamiliar lexical items to prevent disfluencies caused by decoding difficulties.
2. Practice: Prior to the actual recording, participants performed a short practice block consisting of three non-target utterances to familiarize themselves with the scenario-priming protocol.
3. Production: To bridge the gap between scripted reading and spontaneous speech, a scenario-priming protocol was implemented. Instead of reading a static list, stimuli were presented one at a time on a computer screen in a pseudo-randomized order to prevent order effects and modality predictability.

For each trial, participants were presented with a situational context (written in Native Language) designed to elicit a specific communicative intent (e.g., a stern warning vs. a neutral observation). They were instructed to internalize this context before producing the target sentence. This approach aimed to mitigate the list-reading effect and elicit authentic, pragmatically grounded intonational contours. Participants were permitted to self-correct; in cases of hesitations, mispronunciations, or restarts, the final fluent realization was selected for acoustic analysis.

### Annotation

The acoustic data were segmented and annotated using the F_ToBI system (Delais-Roussarie et al., 2015), with initial syllable alignment performed semi-automatically via EasyAlign (Goldman, 2011) and subsequently manually corrected. Two trained linguists specializing in French prosody independently annotated the entire corpus.

Given the specific L1 background of the learners, a stringent multi-dimensional criterion—strictly adhering to the F_ToBI framework (Delais-Roussarie et al., 2015)—was established to identify genuine AP boundaries. Because Mandarin learners frequently produce localized F0 movements (e.g., Tone 4-like falls) on non-final syllables, relying solely on pitch excursions risks severe over-segmentation. Therefore, in accordance with standard French intonational phonology, a boundary was manually coded only if it satisfied converging criteria: (1) Metrical Alignment: The pitch movement aligned with a metrically strong syllable; and (2) Temporal/Rhythmic Cues: The pitch excursion was strictly accompanied by final lengthening or a perceptible rhythmic juncture. F0 fluctuations lacking these obligatory temporal correlates (estimated during manual annotation to represent approximately 10–15% of initial pitch turning points in beginner productions) were categorized as non-rhythmic tonal residues rather than structural AP boundaries. Crucially, because these discarded residues represent excess L1 tonal movements, re-including them in the quantitative analysis would only exponentially increase the learners’ AP counts. Thus, our stringent filtering provides the most conservative test for the Hierarchical Fragmentation Hypothesis (H2), confirming that the persistent over-segmentation observed is a genuine structural deficit rather than a mere artifact of unsuppressed L1 pitch tracking.

Following independent annotation, a cross-checking procedure resolved discrepancies in boundary tone assignment (e.g., distinguishing L% from LL%) and AP segmentation through consensus. Inter-rater reliability was assessed on a randomly selected subset (20%) of the data, yielding a Cohen’s Kappa coefficient of 0.86, indicating excellent agreement (Landis and Gary, 1977).

For quantitative analysis, nuclear contours were classified into three primary categories based on their terminal pitch direction and register, integrating the frameworks of Post (2000), Di Cristo (2016), Martin (2018), and Mertens (2019). These patterns were operationalized via F_ToBI labels as follows: (1) Rising Contours: Including High Rise (H*H%), initiating from the mid-range to a high boundary; and Low Rise (H*HH%), initiating from the lower register; (2) Falling Contours: Including High Fall (L*LL%), characterized by a sharp descent from an elevated pitch peak; and Low Fall (L*L%), confined to the mid-low range; (3) Complex and Flat Contours: Including Rise-Fall (H*L%), Fall-Rise (L*H%), and Flat/Level contours (0%) maintained at either high or low registers.

**Figure 1:**
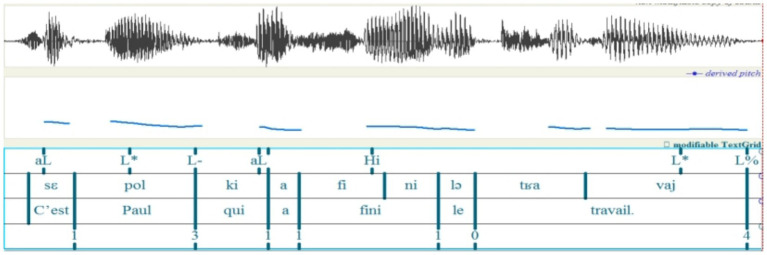
Prosodic configuration and F_ToBI annotation of the sample sentence: “Lavez-vous les mains avant de manger.” (Wash hands before eating).

Pitch register determination was based on F0 peaks from the initial or pre-nuclear accents within each intonational phrase. Figure 1 illustrates these patterns using ToBI annotation.

### Analysis

Acoustic measurements were extracted using the ProsodyPro script (Xu, 2013). To facilitate cross-speaker comparability and account for the logarithmic nature of human pitch perception, all F0 measurements were converted from Hertz to semitones using a fixed reference frequency of 50 Hz.

Four acoustic parameters were calculated to serve as dependent variables: (1) Final pitch span: the magnitude of the terminal rise or fall (in semitones); (2) Final pitch expansion: the standard deviation of F0 on the final syllable, representing local tonal prominence; (3) Final lengthening: the duration of the final syllable relative to the mean syllable duration of the preceding unit, measured as a normalized ratio; and (4) Global pitch span: the difference between the maximum and minimum normalized F0 values across the entire utterance.

Note that while intensity is a correlate of directive force, it was excluded from the analysis to ensure methodological rigor. Given the variation in recording environments (France vs. China) and microphone gain settings, pitch and temporal ratios provide more robust, instrument-independent measures of prosodic contrast.

Statistical analyses were conducted using Linear Mixed-Effects Models (LMM) implemented in R with the *lmerTest* package (Kuznetsova et al., 2017). The four acoustic parameters served as dependent variables in separate models. The fixed effects structure included a three-way interaction between Group [Native (F), Beginner (A), Intermediate (B)], Modality (Statement, Command), and Utterance Length (Short, Long). Following the Keep It Maximal principle (Barr et al., 2013), initial models were constructed with maximal random effect structures, including random slopes for the within-subject factors (Modality and Length). However, these maximal models consistently resulted in boundary (singular) fits, indicating overfitting due to zero-variance estimates in the random slopes (Bates et al., 2015). Consequently, a standard step-down reduction strategy was applied to achieve model parsimony and avoid overfitting, as recommended by Barr et al. (2013). The final models, which successfully converged without singularity, retained only the random intercept for Participant ID. This ensures that the model complexity is empirically supported by the data without inflating Type I error rates. Model fit and effect sizes were evaluated using Marginal (*R^2^_m*) and Conditional (*R^2^_c*) R-squared values via the MuMIn package (Bartoń, 2025). Post-hoc pairwise comparisons for significant interactions were conducted using the *emmeans* package (Lenth, 2024), with Tukey’s HSD adjustment applied to control for family-wise error rates.

## Results

### Prosodic features

**Figure 2:**
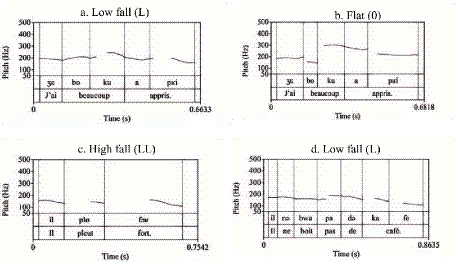
Prototypical prosodic contours of declarative statements produced by native French speakers (group F): **(a)** Low fall (L), **(b)** Flat (0), **(c)** High fall (LL), **(d)** Low fall (L).

**Figure 3:**
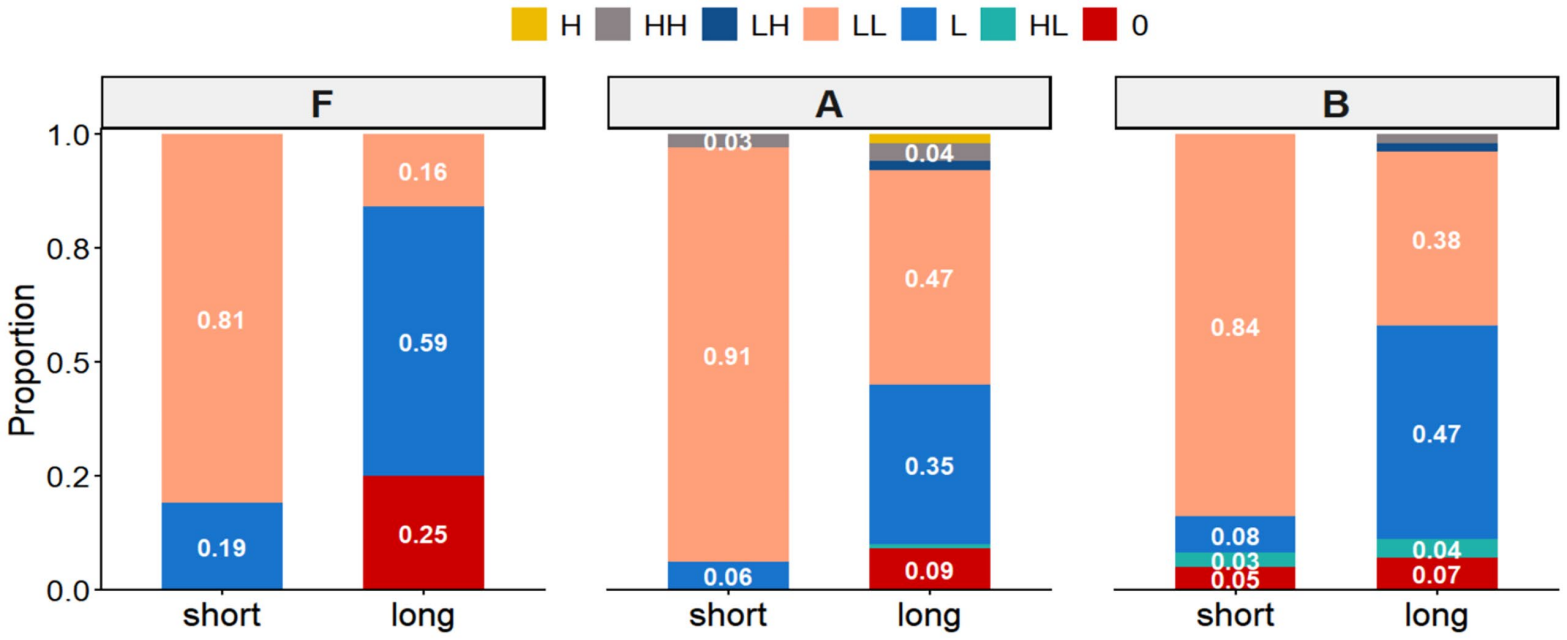
Proportions of different boundary tone configurations observed in statements across group F (native), group A (beginner), and group B (intermediate).

**Figure 4:**
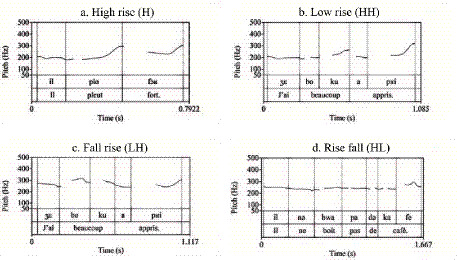
Representative prosodic contours of declarative statements produced by Chinese-speaking learners of French: **(a)** High rise (H), **(b)** Low rise (HH), **(c)** Fall rise (LH), **(d)** Rise fall (HL).

The analysis of declarative statements (comprising short, long, and negative utterances) revealed a distinct interaction between utterance length and proficiency level. In short utterances (e.g., *Il pleut fort*.), the high-falling final contour (LL%) emerged as the canonical realization across all groups. Beginners (Group A) displayed a near-categorical preference for this pattern (0.91), while native speakers (Group F) also employed it predominantly (0.81) (Figures 2c, 3). Intermediate learners (Group B) largely aligned with this trend (0.84), albeit with sporadic instances of flat (0.05) and rise-fall (0.03) variants (Figure 4a).

However, in longer utterances and negations, a significant distributional divergence occurred. Native speakers shifted their primary preference to low-falling nuclear contours (L%) (Figures 2a,d), utilizing the flat contour as a notable secondary option (0.25) (Figure 2b). Intermediate learners successfully mirrored this native-like shift toward the low-falling pattern. In stark contrast, beginners failed to modulate their pitch strategy, retaining the high-falling pattern (LL%) as their primary choice (0.47) even in complex contexts. Notably, the flat contour, a key marker of native continuity in longer phrases, was markedly underutilized by both learner groups.

Regarding non-target contours, deviations were strictly confined to the learner corpus. Rising patterns—including low-rise (HH%), fall-rise (LH%), and high-rise (H%)—were exclusively observed in non-native productions (Figures 4b–d). While these contours were absent in native speech, they constituted a consistent interlanguage feature. Proficiency effects were evident in the distribution of these errors: beginners exhibited a wider repertoire of deviant forms, including the unique use of high-rising contours (0.02), whereas intermediate learners occasionally produced complex rise-fall patterns (HL%) alongside standard low-rises.

**Figure 5:**
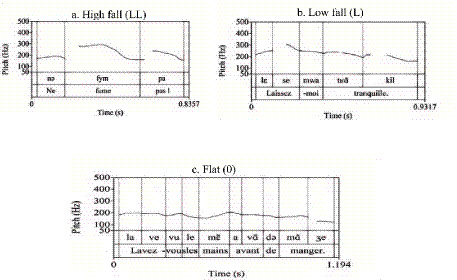
Prototypical prosodic contours of directive commands produced by native French speakers (group F): **(a)** High fall (LL), **(b)** Low fall (L), **(c)** Flat (0).

**Figure 6:**
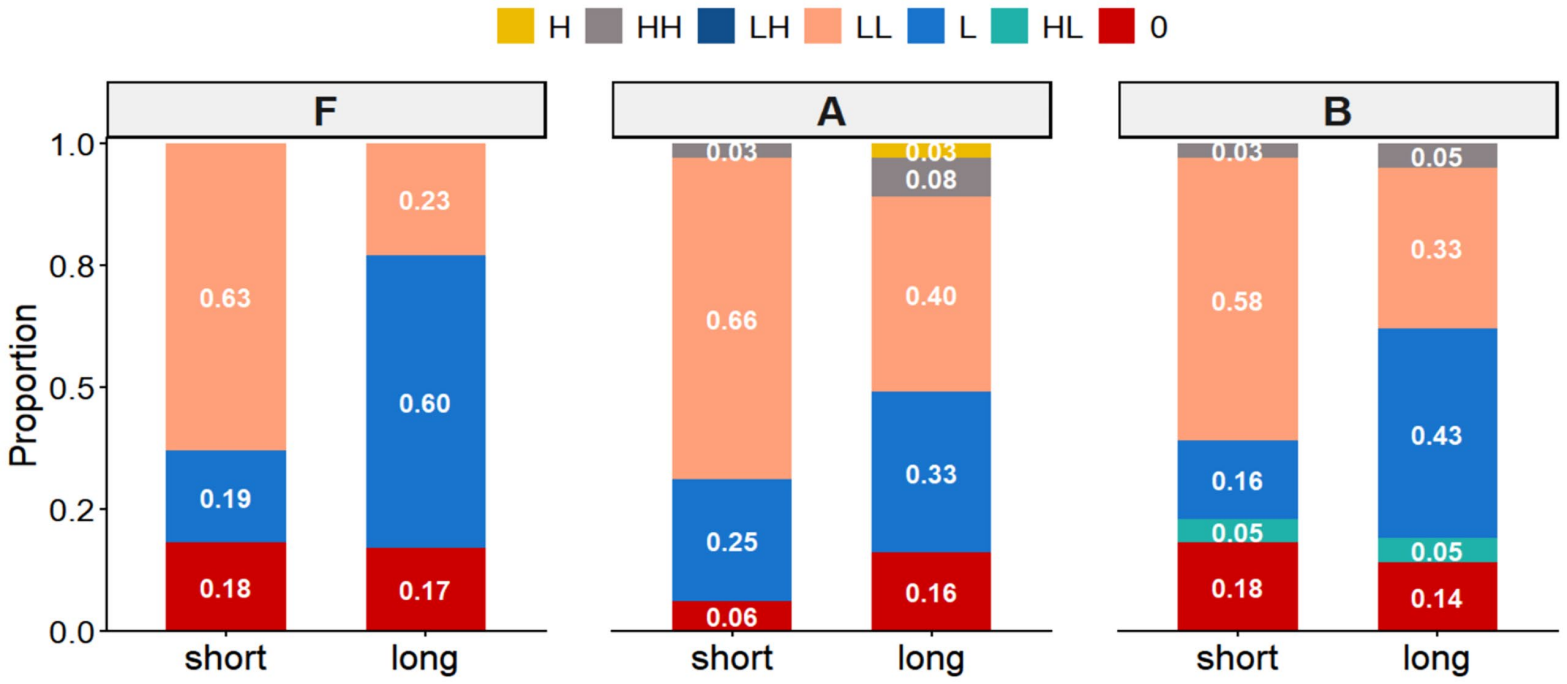
Proportions of different boundary tone configurations observed in commands across Group F (Native), Group A (Beginner), and Group B (Intermediate).

The analysis of commands encompassed short (Ne fume pas!), medium (Laissez-moi tranquille.), and long (Lavez-vous les mains avant de manger.) imperatives. In the short condition, a strong consensus emerged: all groups converged on the high-falling contour (LL%) as the canonical realization (Figures 5a, 6). Secondary variation differed slightly by proficiency; while native speakers and intermediate learners maintained a balance between flat (0%) and low-falling (L%) variants, beginners displayed a distinct preference for low falls over flat contours.

However, syntactic complexity in medium and long imperatives triggered distributional shifts. Native speakers and intermediate learners primarily utilized low-falling contours (L%) (Figure 5b) to encode directive force, although the intermediate group employed this target pattern with notably lower frequency (0.43). For both groups, the high fall (LL%) served as a secondary strategy. In contrast, beginners exhibited a lack of categorical precision, displaying a near-equiprobable distribution between high (LL%) and low (L%) falls. Flat contours remained the third most frequent option across all cohorts.

**Figure 7:**
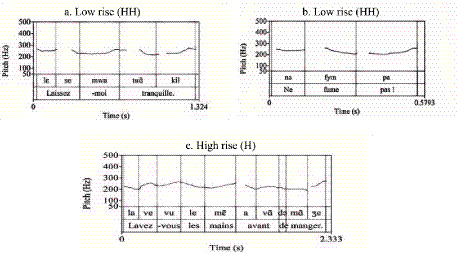
Prosodic contours of commands produced by Chinese-speaking learners of French: **(a)** Low rise (HH), **(b)** Low rise (HH), **(c)** High rise (H).

Distinctive learner-specific features were again restricted to non-native productions. As with statements, rising contours constituted a hallmark of interlanguage prosody. Beginners demonstrated a particular susceptibility to low-rising (Figures 7a,b) and high-rising (Figure 7c) patterns. Intermediate learners, while generally closer to native norms, uniquely produced complex rise-fall contours (HL%). These findings collectively suggest a trajectory of gradual approximation: while intermediate learners successfully internalize the macro-prosodic shape of short commands, the cognitive processing demands of longer directive structures continue to impede their precise prosodic implementation.

### Speech rate and fluency

Speech rate, measured in syllables per second (syll/s), served as a proxy for global oral fluency and processing automaticity (Martin, 2009, 2018). The analysis was restricted to multi-phrasal utterances (containing at least two APs) to ensure sufficient duration for reliable temporal measurement.

**Figure 8:**
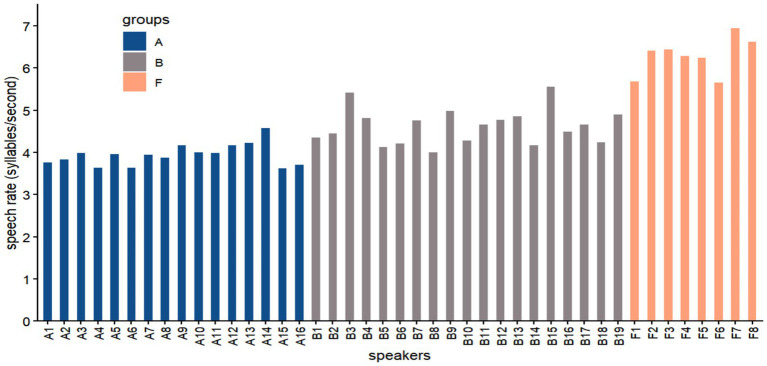
Comparison of individual speech rates (syllables/s) for native speakers (group F) and Mandarin learners at beginner (group A) and intermediate (group B) proficiency levels.

As illustrated in Figures 8, a distinct proficiency-based hierarchy emerged. Native speakers (Group F) exhibited the highest fluency, with a mean rate of 6.5 syll/s. In contrast, learner production was characterized by significantly greater temporal expansion: beginners (Group A) averaged 4.5 syll/s, while intermediate learners (Group B) achieved a moderate increase to 5.4 syll/s.

A Pearson correlation analysis confirmed a robust positive association between L2 proficiency and speech rate among Chinese learners (*r* = 0.694, *p* < 0.001). This strong correlation suggests that the acquisition of L2 prosody is intrinsically linked to temporal automation: as proficiency increases, the cognitive cost of phonological planning decreases, allowing learners to gradually approximate the rhythmic tempo of native speech.

### Segmentation and pre-nuclear stress

The analysis of prosodic phrasing, restricted to utterances containing at least four syllables, revealed a systematic structural fragmentation in learner speech. As detailed in Table 1, native speakers (Group F) maintained cohesive rhythmic units, producing an average of 19.25 APs with a mean span of 3.95 syllables per phrase. In contrast, learner productions were characterized by significant oversegmentation: Beginners (Group A) produced a notably higher frequency of APs (*M* = 22.81) with reduced syllabic spans (*M* = 3.33), while Intermediate learners (Group B) demonstrated only moderate structural integration (M_APs = 21.79, M_Length = 3.49).

**Table 1:** Rhythmic metrics of accentual phrase (AP) division and correlation with French proficiency levels for mandarin learners.

Group	*F*	*B*	*A*
Number of AP	19.25	21.79	22.81
Length of AP (in syllables)	3.95	3.49	3.33
Accuracy (compared to F)		0.70	0.64
Number of AP		*r* = −0.774	*p* < 0.001
Length of AP		*r* = 0.783	*p* < 0.001

Pearson correlation analyses confirmed that rhythmic development is intrinsically linked to L2 proficiency. A strong negative correlation was observed between proficiency and AP frequency (*r* = −0.774, *p* < 0.001), alongside a robust positive correlation for mean AP length (*r* = 0.783, *p* < 0.001). This indicates that as proficiency increases, learners gradually transition from short, fragmented rhythmic chunks toward longer, native-like phrasal planning. Phrasing adequacy scores (relative to native norms) reflected this progression, improving from 0.64 in Group A to 0.70 in Group B.

**Figure 9:**
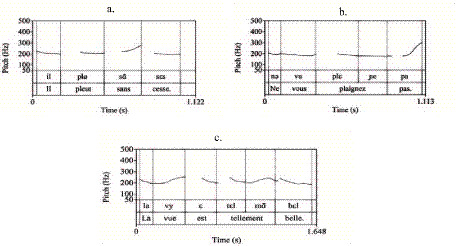
F0 trajectories illustrating characteristic segmentation and accentuation errors: **(a)** accent misplacement, **(b)** monotonous intonation, and **(c)** overemphasized accents.

Qualitatively, the deviation from native rhythmic norms manifested in three primary non-target accentual patterns:

- Metrical Misalignment: The erroneous assignment of pitch prominence to weak syllables [e.g., stress on (sã) as shown in Figure 9a].
- Structural Flatness: The production of monotonous intonation lacking necessary pre-nuclear rhythmic beats, with stress marking confined strictly to the phrase-final syllable (Figure 9b).
- Rhythmic Incongruity: The production of multiple, localized pitch excursions within a single prosodic unit [e.g., dual prominence on (tɛl) and (mã) in Figure 9c], which disrupts the phrase-final focal organization and contributes to an isochronous, disjointed rhythmic pattern.

## Statistical features

In the following LMMs, the unstandardized regression coefficient (β) serves as the primary indicator of effect size. Unlike standardized measures, beta preserves the interpretability of the original acoustic units, representing the predicted deviation in semitones (for pitch parameters) or normalized ratios (for duration) between groups and conditions. Crucially, we distinguish between statistical significance and acoustic relevance. Large coefficient values (e.g., *β* > 2.0 for pitch span) signal substantial acoustic shifts that transcend individual physiological variation (such as micro-prosodic jitter) and fall within the range of perceptual salience. Thus, high β values indicate robust phonological contrasts or significant interlanguage deviations.

### Global and final pitch span

The LMM analysis for Global Pitch Span (Table 2) yielded significant main effects for Group [*F*(2, 710.69) = 37.11, *p* < 0.001] and Modality [*F*(2, 697.78) = 17.43, *p* < 0.001], with the fixed effects explaining 18.6% of the variance (*R^2^*_*m* = 0.186). Crucially, a highly significant Group × Modality interaction was observed [*F*(2, 697.78) = 6.68, *p* = 0.001], confirming that native speakers and learners employ distinct strategies in modulating global pitch range to signal sentence function.

**Table 2:** Linear mixed-effects model results for global and final pitch span (in semitones) across group, modality, and length.

Predictor	Global pitch span	Final pitch span
Group (G)	*F*(2, 710.69) = 37.11, *p* < 0.001	*F*(2, 686.69) = 2.97, *p* = 0.052
Modality (M)	*F*(1, 697.78) = 17.43, *p* < 0.001	*F*(1, 701.70) = 3.66, *p* = 0.056
Length (L)	*F*(1, 697.78) = 17.88, *p* < 0.001	*F*(1, 701.70) = 34.70, *p* < 0.001
G × M	*F*(2, 697.78) = 6.68, *p* = 0.001	*F*(2, 701.70) = 0.99, *p* = 0.371
G × L	*F*(2, 697.78) = 1.89, *p* = 0.151	*F*(2, 701.70) = 2.94, *p* = 0.054
M × L	*F*(1, 697.78) = 4.20, *p* = 0.041	*F*(1, 701.70) = 0.15, *p* = 0.702
G × M × L	*F*(2, 697.78) = 0.02, *p* = 0.981	*F*(2, 701.70) = 0.84, *p* = 0.431
*R^2^* (marginal)	0.186	0.063

Notably, the three-way interaction (Group × Modality × Length) was non-significant [*F*(2, 697.78) = 0.02, *p* = 0.981]. While non-significant results must be interpreted with caution—especially given the current sample size which may limit statistical power to detect subtle micro-developmental shifts—this finding is nonetheless theoretically indicative. It suggests that the phenomenon of prosodic merging (the learners’ persistent failure to differentiate commands from statements) is unlikely to be merely an artifact of sentence length. Rather, the pervasive lack of significance, coupled with the inflated Standard Errors observed in learner groups, points toward a highly stochastic interlanguage system where tonal interference acts as a robust hurdle across varying rhythmic demands.

**Figure 10:**
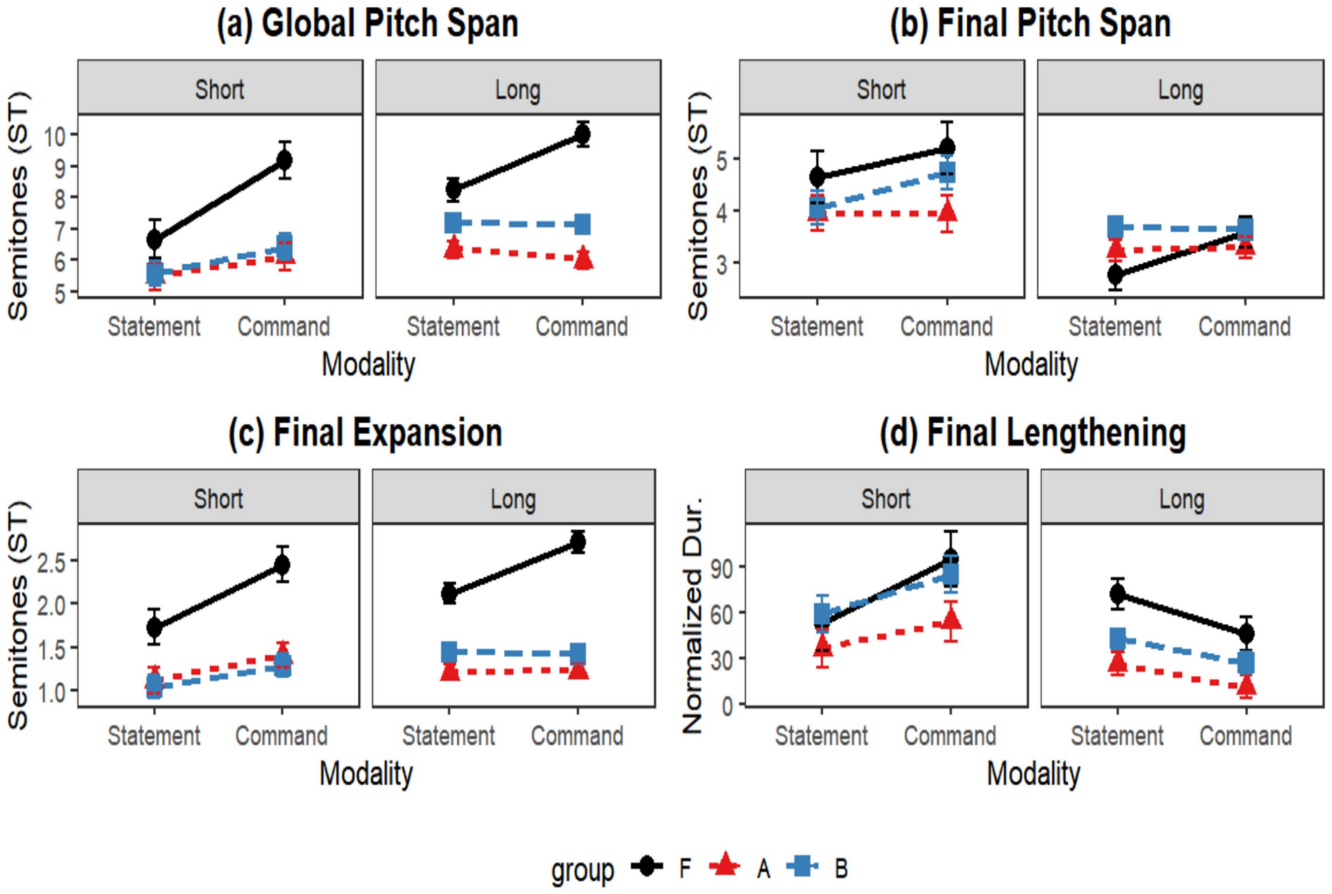
Interaction effects of group, modality, and length on the four prosodic dimensions: **(a)** global pitch span, **(b)** final pitch span, **(c)** final expansion, and **(d)** final lengthening.

As illustrated in Figure 10a, native speakers (Group F) systematically expanded their global pitch envelope for commands. Within-group contrasts (Table 3) confirmed significant expansion in both short (*β* = −2.53, *p* = 0.002) and long utterances (*β* = −1.78, *p* < 0.001). Conversely, neither the Beginner (Group A) nor the Intermediate group (Group B) achieved significant prosodic differentiation between modalities (all *p* > 0.05). *Post hoc* pairwise comparisons (Table 4) quantified this deficit: in the critical Command-Long condition, beginners undershot native targets by an average of 4.01 semitones (*p* < 0.001), and intermediate learners by 2.90 semitones (*p* < 0.001). While Group B showed significant improvement over Group A in long statements (*β* = −0.86, *p* = 0.011), this progression was absent in commands, which provides empirical evidence for the prosodic merging hypothesis, irrespective of proficiency level.

**Table 3:** Within-group modality contrasts (statement vs. command) across proficiency levels and utterance lengths.

Group	Length	β	SE	*t*	*p*
F	Short	−2.53	0.82	−3.10	0.002
	Long	−1.78	0.45	−3.92	<0.001
A	Short	−0.63	0.58	−1.10	0.274
	Long	0.33	0.32	1.02	0.309
B	Short	−0.87	0.53	−1.64	0.102
	Long	−0.08	0.30	0.27	0.790

β and SE values for Pitch Span/Expansion are in semitones; values for Lengthening are normalized ratios.

**Table 4:** Comparisons of global pitch span and final pitch span between native speakers (F) and learner groups (A,B).

Condition	Contrast	Global pitch span				Final pitch span			
		β	SE	*t*	*p*	β	SE	*t*	*p*
ST-short	F-A	1.18	0.71	1.66	0.222	0.69	0.59	1.17	0.470
	F-B	1.11	0.69	1.60	0.246	0.59	0.58	1.02	0.567
	A-B	−0.07	0.56	−0.13	0.991	−0.11	0.46	−0.23	0.971
CO-short	F-A	3.08	0.71	4.32	<0.001	1.26	0.59	2.12	0.086
	F-B	2.77	0.69	3.99	<0.001	0.46	0.58	0.80	0.700
	A-B	−0.30	0.56	−0.55	0.847	−0.79	0.46	−1.72	0.200
CO-short	F-A	1.91	0.39	4.92	<0.001	−0.48	0.32	−1.50	0.293
	F-B	1.04	0.38	2.74	0.017	−0.93	0.31	−2.96	0.009
	A-B	−0.86	0.30	−2.89	0.011	−0.45	0.25	−1.81	0.011
CO-long	F-A	4.01	0.42	9.63	<0.001	0.28	0.35	0.82	0.688
	F-B	2.90	0.41	7.11	<0.001	−0.05	0.34	−0.16	0.986
	A-B	−1.11	0.32	−3.45	0.002	−0.34	0.27	−1.26	0.417

β and SE values for Pitch Span/Expansion are in semitones; values for Lengthening are normalized ratios.

Furthermore, an analysis of variance reveals that the non-significant modality contrasts in learner groups were accompanied by inflated Standard Errors (SE) compared to native speakers (Table 4). For instance, in the Command-Long condition, learner SE values were substantially higher. This heightened variability suggests that while native speakers occupy a tightly clustered, high-precision phonetic space, learner productions are characterized by considerable inter-speaker inconsistency and interlanguage flux.

Regarding Final Pitch Span, the main effect of Length was dominant [*F*(1, 701.70) = 34.70, *p* < 0.001], whereas the effect of Group reached only marginal significance (*p* = 0.052). Within-group contrasts (Table 3) revealed that only native speakers significantly adjusted their final pitch span for modality in long sentences (*β* = −0.93, *p* = 0.009). For learners, the terminal rise or fall was primarily governed by physiological constraints (declination over length) rather than the functional requirement of encoding directive force.

It is critical to highlight the disparity in explanatory power between the two models. The Marginal R^2^ for Final Pitch Span (*R^2^_m* = 0.063) was markedly lower than that for Global Pitch Span (*R^2^_m* = 0.186). While differences in explained variance do not inherently prove a cognitive hierarchy, this statistical discrepancy suggests a bifurcation of systematicity in L2 acquisition: whereas macro-level intonational envelopes (Tier 2) are increasingly governed by systematic group-level patterns, local pitch targets on the nuclear syllable (Tier 3) remain characterized by high stochasticity. At this micro-level, learner productions have not yet stabilized into categorical targets, rendering them highly susceptible to idiosyncratic phonetic strategies and micro-prosodic perturbations. This unsystematic variability supports the notion that while learners are successfully automating the structural frame of French intonation, the precise acoustic reshaping of the nuclear syllable remains a site of phonetic uncertainty.

### Final expansion and final lengthening

The analysis of Final Expansion, serving as a proxy for nuclear prominence, revealed a robust main effect of Group (Table 5) [*F*(2, 701.73) = 57.00, *p* < 0.001, *R^2^_m* = 0.228] and a significant Group × Modality interaction [*F*(2, 699.25) = 4.51, *p* = 0.011]. Native speakers systematically amplified F0 excursion on the final syllable to encode directive force, particularly in long utterances (Figure 10c).

**Table 5:** Linear mixed-effects model results for final expansion and final lengthening across group, modality, and length.

Predictor	Final expansion	Final lengthening
Group (G)	*F*(2, 701.73) = 57.00, *p* < 0.001	*F*(2, 665.25) = 8.49, *p* < 0.001
Modality (M)	*F*(1, 699.25) = 17.41, *p* < 0.001	*F*(1, 701.65) = 0.50, *p* = 0.479
Length (L)	*F*(1, 699.25) = 6.23, *p* = 0.013	*F*(1, 701.65) = 15.23, *p* < 0.001
G × M	*F*(2, 699.25) = 4.51, *p* = 0.011	*F*(2, 701.65) = 0.08, *p* = 0.924
G × L	*F*(2, 699.25) = 2.67, *p* = 0.070	*F*(2, 701.65) = 0.88, *p* = 0.413
M × L	*F*(1, 699.25) = 2.39, *p* = 0.123	*F*(1, 701.65) = 12.73, *p* < 0.001
G × M × L	*F*(2, 699.25) = 0.07, *p* = 0.930	*F*(2, 701.65) = 0.52, *p* = 0.598
*R^2^* (marginal)	0.228	0.079

In contrast, learner productions were characterized by a marked pragmatic undershoot. Post-hoc comparisons (Table 6) confirmed that in the critical Command-Long condition, deviations from native norms were substantial: Beginners (Group A) exhibited the most severe hypo-articulation (F-A: *β* = 1.80, *p* < 0.001), followed closely by Intermediate learners (F-B: *β* = 1.37, *p* < 0.001). Crucially, while Group B demonstrated significant improvement over Group A in long statements (*β* = −0.53, *p* = 0.022), no such developmental progression was observed for commands (*β* = −0.43, *p* = 0.175). This stagnation indicates that the acoustic marking of insistence—requiring abrupt terminal pitch falls—remains a persistent hurdle for Mandarin speakers, resisting automation even at higher proficiency levels.

**Table 6:** *Post hoc* pairwise comparisons of final expansion and lengthening between native speakers (F) and learner groups (A,B).

Condition	Contrast	Final expansion				Final lengthening			
		β	SE	*t*	*p*	β	SE	*t*	*p*
ST-short	F-A	0.22	0.26	0.85	0.673	0.12	0.08	1.50	0.291
	F-B	−0.15	0.25	−0.60	0.821	0.08	0.08	1.00	0.578
	A-B	−0.37	0.22	−1.68	0.214	−0.04	0.07	−0.57	0.836
CO-short	F-A	0.78	0.26	3.00	0.008	0.24	0.08	3.00	0.008
	F-B	0.42	0.25	1.68	0.214	0.14	0.08	1.75	0.188
	A-B	−0.36	0.22	−1.64	0.230	−0.10	0.07	−0.43	0.326
ST-short	F-A	0.30	0.25	1.20	0.453	0.35	0.09	3.89	<0.001
	F-B	−0.23	0.24	−0.96	0.602	0.28	0.08	3.50	0.001
	A-B	−0.53	0.20	−2.65	0.022	−0.07	0.07	−1.00	0.578
CO-long	F-A	1.80	0.31	5.81	<0.001	0.18	0.08	2.25	0.045
	F-B	1.37	0.30	4.57	<0.001	0.06	0.08	0.75	0.734
	A-B	−0.43	0.24	−1.79	0.175	−0.12	0.07	−1.71	0.202

β and SE values for Pitch Span/Expansion are in semitones; values for Lengthening are normalized ratios.

Furthermore, the statistical non-significance of modality contrasts within learner groups is corroborated by inflated Standard Error (SE) values (Table 6). This suggests that the lack of a group-level effect is not merely due to a uniform inability to produce pitch changes, but rather stems from high idiosyncratic variation. Learner strategies appeared stochastic: while some attempted pitch excursions, others maintained flat contours, resulting in an overall statistical plateau characteristic of unstable interlanguage systems.

Regarding the temporal dimension, Final Lengthening yielded a significant main effect of Group [*F*(2, 665.25) = 8.49, *p* < 0.001] and a strong Modality × Length interaction [*F*(1, 701.65) = 12.73, *p* < 0.001]. Native speakers consistently employed robust phrase-final lengthening to demarcate structural boundaries (Figure 10d). Learners, however, struggled to replicate this temporal cue. In long statements (Table 6), both groups significantly undershot native duration targets (F-A: *β* = 0.35, *p* < 0.001; F-B: *β* = 0.28, *p* = 0.001). Interestingly, a subtle developmental shift emerged in long commands: while beginners differed significantly from native speakers (*β* = 0.18, *p* = 0.045), intermediate learners achieved marginal convergence (*p* = 0.734).

Collectively, these findings highlight a multi-dimensional deficit at the right edge of the prosodic phrase. The combination of temporal deviations (insufficient lengthening) and melodic flatness (lack of expansion) confirms that Chinese learners struggle to integrate rhythmic and intonational cues to mark French APs. This failure to align pitch targets with durational boundaries results in a prosodic structure that native listeners are likely to perceive as structurally monotonous and pragmatically ambiguous.

### Summary of developmental progression

The integration of post-hoc pairwise comparisons (Tables 4, 6) and within-group contrasts (Table 3) delineates a distinctly bifurcated acquisition trajectory. On one hand, Intermediate learners (Group B) demonstrated significant convergence toward native norms in the Statement modality. Their ability to appropriately expand global pitch ranges, particularly in longer declarative utterances, suggests a successful automation of macro-prosodic structural planning. On the other hand, this structural competence did not transfer to the pragmatic domain. Both learner groups reached a persistent developmental plateau in the production of Commands. The lack of significant modulation in Global Pitch Span and Final Expansion (as evidenced by the null results in Table 3) indicates that learners did not systematically utilize pitch as a cue for illocutionary intent. This asymmetry underscores a critical finding: for Mandarin speakers, L2 prosodic acquisition is not uniform but functional-specific. While the prosody required for structural demarcation (declaratives) is gradually mastered, the cognitive re-weighting of cues necessary to encode French directive force remains a site of significant typological resistance.

## Discussion

The present study elucidates the developmental trajectory of L2 French prosody among Mandarin learners, uncovering a distinct asymmetry between the mastery of structural boundaries and the encoding of communicative intent. By probing the acoustic realization of modality across varying degrees of linguistic complexity, our findings isolate the specific functional and typological constraints that govern the tone-to-intonation transition. The following discussion synthesizes these acoustic results to evaluate the four core hypotheses (H1–H4), ultimately framing the observed “structure-first, pragmatics-later” progression within the proposed Modality-Prosody Acquisition Hierarchy (MPAH), a theoretical model of the processing demands inherent in L2 prosodic integration.

### Prosodic merging and functional constraints on modality encoding (H1 and H4)

The results substantiate the Prosodic Merging Hypothesis (H1): Mandarin learners failed to mobilize pitch modulation as a robust functional marker of modality. This aligns with evidence regarding the persistent crosstalk between L1 tone and L2 intonation (Zahner-Ritter et al., 2022). As evidenced by the non-significant interaction contrasts (Table 3), learners defaulted to a generalized, declarative-like prosodic template across both modality conditions. This behavior signals a clear Syntactic-Prosodic Dissociation: while learners have mastered the Tier 1 (lexical/syntactic) markers of modality (e.g., subject deletion), they encounter a persistent functional integration deficit at Tier 3 (micro-prosodic encoding). This suggests that the deficit is not a product of communicative negligence, but rather a structural inability to reallocate pitch resources for pragmatic functions, consistent with the hypothesis that L2 production under high communicative pressure requires significant computational resources to resolve competing linguistic representations (though these mechanisms remain theoretical inferences in the absence of direct cognitive measures).

Beyond global span reduction, acoustic analyses revealed systematic deviations in nuclear configurations. While native speakers utilized low-falling contours (L*L%) to signal finality in longer utterances, beginners over-relied on high falls (L*LL%), and intermediate learners achieved only partial acquisition. Notably, the emergence of rising contours in learner commands—a pattern unattested in native productions—suggests a complex interaction of L1 transfer (Mandarin question intonation; Shi, 2013), overgeneralization of short-utterance patterns (Delais-Roussarie et al., 2015), and compensatory strategies for linguistic uncertainty. For Mandarin learners, the rigid L1 tonal framework appears to impede the effective decoupling of lexical pitch from phrasal modulation, corroborating the L2 Intonation Learning Theory (Mennen, 2015).

This merging phenomenon is further constrained by the Complexity-Modality Trade-off (H4). As predicted, the gap between native and learner prosody peaked in complex conditions (e.g., *Lavez-vous les mains avant de manger*.). Viewed through the lens of the Competition Model (MacWhinney, 1987) and Cognitive Load Theory (Sweller, 1988), encoding directive force in long structures may impose a dual processing demand: simultaneous syntactic planning and prosodic re-weighting. While this study does not provide direct behavioral measures of cognitive capacity, we hypothesize that the increased computational demand contributes to the reduction of pragmatic prosodic markers, thereby inducing phonetic undershoot. Specifically, navigating complex utterances likely taxes processing resources during speech production, making it more difficult for learners to reallocate L1 tonal habits while executing fine-grained L2 pitch modulation. Under such constraints, learners appear to prioritize structural stability, a strategy that necessitates a trade-off where pragmatic precision is sacrificed.

Finally, the statistical non-significance of learner modality contrasts (*p* > 0.05) provides a window into the stochastic nature of the interlanguage system. The elevated Standard Errors (SE) reported in section 3.4 suggest that learner productions have not yet systematized into a coherent phonological grammar, in contrast to the categorical stability observed in native speakers. However, a subtle developmental trend is visible: the reduction in variability from Beginners (SE = 0.42) to Intermediate learners (SE = 0.41) in the Command-Long condition suggests the onset of stabilization. This persistent variability confirms that while the structural frame is being automated, pragmatic prosody remains a site of phonetic and functional uncertainty, sensitive to the fluctuating allocation of processing resources.

### Hierarchical fragmentation and prosodic percolation (H2)

The analysis of rhythmic organization substantiates the Hierarchical Fragmentation Hypothesis (H2). As evidenced by the significant increase in AP frequency and reduction in span length (Table 1), learner productions were characterized by systematic oversegmentation. Crucially, this fragmentation persists even after rigorously filtering out non-rhythmic tonal residues during annotation. This methodological control demonstrates that the observed segmentation is not a byproduct of acoustic jitter or L1 pitch tracking, but reflects a structural breakdown in the planning of L2 prosody.

Within the framework of the Prosodic Hierarchy (Nespor and Vogel, 1986), native French prosody relies on a successful mapping from terminal nodes (the Phonological Word) to higher-level phrasal constituents [APs and Intonational Phrases (IPs)] (Jun and Fougeron, 2000). Native speakers treat the AP as a cohesive planning unit, reserving substantial pitch movements exclusively for the right boundary to signal modality.

In contrast, Mandarin learners appear to process each Phonological Word as an independent prosodic domain. We characterize this phenomenon as a failure of prosodic percolation: the learner’s inability to suppress syllable-level prominence in favor of higher-level phrasal targets. For these speakers, the L1 syllable-timed rhythmic framework imposes a rigid one-to-one (isomorphic) mapping constraint, causing the prosodic hierarchy to decompose into fragmented rhythmic chunks. This finding resonates with Michelas and D’Imperio (2010) observations regarding the late acquisition of phrasal integration in L2 French.

It is important to address a potential methodological concern: does the filtering of non-rhythmic tonal residues artificially create an impression of structural flatness? We argue the contrary. These identified pitch fluctuations—representing unsuppressed L1 lexical tones that fail to align with the L2 prosodic hierarchy—are distinct from genuine AP boundaries. If these tonal residues were coded as valid APs, the degree of Hierarchical Fragmentation (H2) reported in Table 1 would be even more severe, revealing an even greater degree of rhythmic instability. By excluding these residues, we demonstrate that even when we strip away the most obvious layer of L1 tonal interference, the underlying L2 prosodic structure remains significantly more fragmented than that of native speakers. This confirms that the deficit is not merely an accumulation of surface errors, but a fundamental failure in phrasal planning.

Despite this initial fragmentation, the data reveal a clear path toward integration. The strong correlations between proficiency and phrasing metrics (*r* = −0.774 for AP count; *r* = 0.783 for AP length) indicate that as phonological automation increases, learners gradually reduce the frequent, non-native pitch resets that result in disjointed rhythmic phrasing. However, this rhythmic recovery is only one facet of the acquisition process; it interacts complexly with the persistent deficits in pitch modulation, a disassociation we explore through the MPAH model in the following section.

### Acoustic imbalance and the MPAH model (H3)

The acquisition trajectory of Mandarin learners is characterized by a significant acoustic asynchrony, confirming Hypothesis 3. This developmental imbalance is consistent with McClellan (2024) multifactorial model of L2 prosody, which highlights the divergent developmental rates across different intonational dimensions, such as phrasal grouping and pitch range expansion. Building on the functional constraints identified in Section 4.1, we formalize this progression as the MPAH. Unlike purely descriptive models, the MPAH posits that the stratification of L2 prosodic development is governed by the structural and functional distance between the target system and the L1 phonological framework within the Prosodic Hierarchy (Nespor and Vogel, 1986).

Aligning with the multisystemic hypothesis of L2 prosody (Li and Post, 2014)—which suggests that rhythmic and intonational properties follow distinct acquisition timelines—the MPAH is organized into three cumulative tiers:

Tier 1: Lexical and Syntactic Grounding. This stage focuses on overt morphological and syntactic markers (e.g., imperative verb forms, subject deletion). Our results indicate that both learner groups have stabilized this stage, producing grammatically accurate structures.

Tier 2: Macro-Prosodic Expansion (IP-Level Planning). This involves organizing the global intonational envelope of the IP. Intermediate learners successfully expanded their Global Pitch Span to match native targets. Theoretically, we interpret Tier 2 as prosody for demarcation. Because the IP boundary constitutes a high-level node in the Prosodic Hierarchy (Jun and Fougeron, 2000), it functions as a primary structural scaffolding that is relatively computationally efficient for learners compared to lower-level prosodic targets.

Tier 3: Functional Boundary Reshaping (Nuclear Mapping). This final stage requires the precision mapping of IP-final boundary tones (L%) and nuclear prominence to encode directive force. Both learner groups remain stalled here, failing to move beyond structural defaults to implement the sharp F0 falls and significant Final Expansion required for commands.

The processing bottleneck at Tier 3 can be interpreted through the Resolution-Load Hypothesis: macro-prosodic envelopes (Tier 2) require low-resolution (coarse-grained) planning, whereas nuclear reshaping (Tier 3) demands high-resolution (fine-grained) integration of pitch, duration, and intensity on a single syllable. For Mandarin learners, this Tier 3 requirement likely exceeds their current processing capacity. Consequently, they prioritize structural stability over pragmatic nuance.

This micro-level failure highlights the inherent non-linearity and stochasticity of L2 prosodic development. The low explanatory power (*R^2^* = 0.063) observed for Final Pitch Span reflects high inter-speaker variability and stochastic production patterns at the micro-prosodic level. While learners successfully automate the structural frame (Tier 2), they have yet to stabilize a systematic phonological grammar for local modality, rendering Tier 3 a persistent developmental challenge.

While the current MPAH framework is formulated based on the specific typological dynamics of the French-Mandarin dyad, its tiered structure—prioritizing macro-structural demarcation over micro-pragmatic encoding—offers a testable hypothesis regarding L2 cognitive economy. This progression aligns with recent psycholinguistic evidence; for instance, while Zhao et al. (2025) demonstrated that cognitive abilities are crucial for comprehending focus prosody, our findings hypothesize that in L2 production, structural automation (Tier 2) may serve as a similar cognitive prerequisite before learners can allocate resources to high-resolution pragmatic modulation (Tier 3). However, because this model is derived from a single L1-L2 pairing involving a tone-to-intonation transition, further empirical validation is required. Future research incorporating independent cognitive measures and diverse typological pairs (e.g., Germanic-to-Romance) is necessary to determine whether this structure-first, pragmatics-later trajectory is a generalized cross-linguistic phenomenon or a specific artifact of L1 tonal constraints.

Thus, the MPAH offers a cross-linguistic roadmap: the automation of a structural foundation (Tier 2) serves as a prerequisite for fine-grained pragmatic modulation (Tier 3). This hierarchical distinction aligns with the modality-neutral prosodic framework (Prieto et al., 2025), which argues that phrasal grouping and the marking of pragmatic intent are distinct, high-level components of a learner’s prosodic grammar. Future research across diverse typological pairs (e.g., Germanic-to-Romance) is required to validate whether the prosodic merging observed here is a general byproduct of typological distance or a specific artifact of the tone-to-intonation transition.

While the tiered structure of the MPAH attributes this developmental asynchrony to a hierarchical structural dependency, it is critical to evaluate this framework against alternative explanations. First, regarding motor difficulty, one might argue that executing a sharp nuclear fall (Tier 3) is simply more articulatorily demanding than expanding a global pitch envelope (Tier 2). However, Mandarin learners routinely produce isomorphic sharp falls (Tone 4) in their L1, indicating that the deficit is not a pure motoric constraint but a functional reallocation problem. Second, concerning perceptual salience, it could be hypothesized that micro-prosodic nuclear reshaping is less perceptually salient to learners than macro-prosodic global span. While plausible, the frequent inadvertent production of high-falling contours in declarative statements (“lexical leakage”) suggests that learners perceive and possess the acoustic category, yet fail to map it intentionally to pragmatic functions. Finally, the lower explanatory power (*R^2^*_m) at the micro-level confirms that local nuclear targets are inherently more susceptible to idiosyncratic variation than global phrasing. By adjudicating between these competing accounts, the MPAH framework posits that the observed statistical variability and functional misallocation are symptoms of an underlying asynchronous processing constraint: structural automation (Tier 2) must be secured before learners can reliably allocate resources to high-resolution pragmatic mapping (Tier 3).

### Functional decoupling and the dynamics of tonal interference

A significant theoretical puzzle emerges from the data: why do Mandarin learners exhibit prosodic merging and final undershoot in French commands, despite the existence of a high-falling tone (Tone 4) in their L1 that is acoustically isomorphic to the French imperative contour (L*L%)? According to a surface-based Contrastive Analysis, Tone 4 should facilitate the acquisition of directive intonation. However, our results suggest a more complex process of “functional decoupling,” wherein the heavy functional load of lexical tone competes with intonational resources (Liang and van Heuven, 2009). For Mandarin learners, the rigid L1 tonal framework appears to block the flexible reallocation of pitch for phrasal-level pragmatic functions, *which aligns with the dimensional acquisition constraints proposed by Mennen (2015). This difficulty is further elucidated by recent computational reviews (e.g., Huang et al., 2026), which emphasize that Mandarin tones are processed as complex, time-evolving F0 trajectories; such deeply entrenched motoric patterns for local pitch movement likely hinder the flexible reallocation of F0 for phrasal-level pragmatic functions.

The data presents a compelling paradox: as noted in Section 3.1, beginners frequently produce high-falling contours (L*LL%) in long declarative statements, yet fail to deploy this same contour to signal commands. This discrepancy reveals that while the motoric capacity to produce a Tone 4-like fall is available, it is functionally misallocated. We argue that the high falls observed in declaratives represent lexical leakage—instances where low cognitive monitoring allows L1 tonal contours to surface inadvertently on individual words. Conversely, the production of a Command imposes a significantly higher computational demands (Hypothesis 4), requiring the simultaneous integration of syntactic planning and pragmatic signaling. Faced with this high-stakes task, learners appear to adopt a defensive strategy of active over-suppression. While our acoustic data cannot directly confirm underlying neural mechanisms, this phonetic pattern is theoretically consistent with the principles of bilingual inhibitory control (Green, 1998). Recent empirical evidence (Lee et al., 2025) further suggests that the dynamics of such inhibition are highly sensitive to production modality, where top-down planning (similar to our Command condition) hypothetically increases the activation–inhibition burden compared to more bottom-up or automated tasks. Within the unified framework of inhibitory control (Munakata et al., 2011), this phenomenon can be modeled as a competitive process: the goal of maintaining a cohesive L2 rhythmic structure exerts broad inhibitory pressure on local pitch variations, effectively silencing the necessary pragmatic contours. Although these cognitive models are typically applied to lexico-semantic or syntactic control, their application here provides a useful theoretical heuristic for understanding the inhibitory dynamics inherent in L2 prosodic production.

This inhibitory strategy stems from a fundamental domain mapping mismatch: learners must re-map the high-falling contour from its L1 status as a syllable-tethered lexical property to an L2 phrasal boundary tone (L%) or emphatic accent. This mismatch is intensified by conflicts in prosodic alignment (Lu and Jin, 2016): whereas French intensification requires reshaped contours distributed across the phrase, the Mandarin tonal framework prioritizes stable pitch targets for every lexical item.

Consequently, as learners transition from a syllable-timed to a phrase-timed system, they engage in generalized inhibitory control mechanisms. In an effort to avoid the rhythmic fragmentation characteristic of L1 Mandarin and the accidental production of tonalized F0 excursions on non-final syllables, learners appear to rigidly constrain their global pitch range to ensure native-like declarative declination. This compensatory prosodic strategy, while effectively maintaining structural integrity, results in pragmatic undershoot: in prioritizing the rhythmic framework, learners unintentionally dampen the nuclear pitch intensification necessary to encode directive force. Thus, the high-falling contour remains accessible as an inadvertent lexical emergence (or leakage) but is not yet available as a consistent communicative resource. These findings suggest that L2 prosody acquisition involves more than the transfer of acoustic shapes; it entails a complex re-weighting of the interface between phonology and pragmatics. By prioritizing rhythmic structure (phrase-timed grouping) over modal intensification, learners treat the specific intonation of commands as a late-acquired feature at the prosody-pragmatics interface. Indeed, the cognitive cost associated with processing tonal patterns, even in L1 acquisition (Lin et al., 2026), underscores the robust nature of the Mandarin tonal template, which appears to impede the functional decoupling of F0 from syllable-level targets in French phrasal intonation.

Finally, we must address the alternative hypothesis that the observed pragmatic stagnation stems from a perceptual deficit—specifically, the Stress Deafness often attributed to speakers of fixed-stress languages (Dupoux et al., 1997)—rather than a functional processing constraint. While this study did not include a perception task, internal evidence from the production data argues against a purely perceptual explanation. As discussed above, the prevalence of lexical leakage (high falls in declaratives) confirms that the acoustic category of the high fall is not only perceptually salient to learners (mapping onto L1 Tone 4) but also motorically available. If the deficit were purely perceptual, learners would likely fail to produce this contour across all modalities. The fact that they successfully execute high falls as unintentional lexical errors, yet systematically fail to recruit them for intentional pragmatic marking in commands, strongly suggests that the resource allocation failure occurs at the level of functional mapping and executive resource allocation. This aligns with the refined Inhibitory Control framework (Declerck and Koch, 2023), which posits that local-level inhibitory effects are integral to resolving interference in bilingual speech production, albeit subject to ongoing scrutiny regarding the cross-task replicability of these inhibitory effects.

### Fluency and temporal progression

Complementing the analysis of nuclear configurations, the investigation of temporal features reveals a profound typological gap. Speech rate served as a robust indicator of processing efficiency and procedural fluency (Martin, 2009, 2018). The strong positive correlation between proficiency and oral fluency (*r* = 0.694) underscores that achieving native-like tempo is not merely a motoric task but a prosodic planning challenge. Following Segalowitz (2010), we interpret this lag as a deficit in procedural automaticity: lower-proficiency learners have not yet proceduralized L2 prosodic planning, resulting in temporal expansion associated with the high processing load of online prosodic monitoring.

Crucially, the analysis of Final Lengthening exposes the resilience of L1 rhythmic interference. The persistent undershoot of phrase-final duration among learners—standing in sharp contrast to the robust structural lengthening employed by native speakers—confirms that the acquisition of French phrase-timed rhythm lags significantly behind other prosodic competencies.

These findings lend critical empirical support to the Acoustic Imbalance Hypothesis (H3). While intermediate learners demonstrated some capacity to manipulate utterance-level pitch trajectories (as seen in Tier 2), they failed to integrate the necessary durational cues to mark phrasal boundaries. This suggests a specific developmental hierarchy: learners prioritize the macro-melodic dimension (Global F0) while the temporal-structural framework (duration) remains a late-acquired feature. This delay is likely driven by the increased processing demand of managing duration at phrasal boundaries, a function that conflicts with the syllable-based rhythmic constraints of the L1.

Synthesizing these rhythmic, melodic, and temporal deviations, it becomes clear that mere exposure is insufficient. L2 prosody acquisition requires a structured, multi-dimensional intervention to explicitly bridge these typological divides. This necessity informs the pedagogical implications proposed below.

## Conclusion

This study establishes that the acquisition of L2 French prosody by Mandarin learners is governed by a fundamental functional split between structural demarcation and pragmatic communication. The central theoretical contribution of this work is the MPAH, which posits a tiered developmental sequence where the automation of macro-prosodic frames serves as a prerequisite for the mastery of micro-prosodic pragmatic encoding. Our findings confirm that while learners successfully transition from syllable-timed to phrase-timed rhythmic organizations (Tier 2), they reach a persistent plateau in the nuclear reshaping required for directive force (Tier 3). Consequently, the interlanguage system remains structurally sound but pragmatically restricted.

Beyond the specific typological dynamics of the Mandarin-French dyad, these results offer profound insights into the processing demands of L2 production. The observed structure-first, pragmatics-later trajectory suggests that the human language processor prioritizes the construction of stable syntactic-prosodic skeletons before attending to the pragmatic encoding of illocutionary intent. This bifurcation refines the Prosodic Transfer Hypothesis: L1 interference is not a blanket effect, but a selective constraint that specifically targets the most resistant aspect of intonation—the communicative reshaping of nuclear configurations under high cognitive load.

Pedagogically, these theoretical insights necessitate a paradigm shift toward a tiered instructional framework that respects the cognitive processing constraints of L2 learners, prioritizing structural automation before pragmatic refinement.

To contextualize these findings, several methodological constraints warrant discussion. First, regarding acoustic parameters, the exclusion of intensity metrics—necessitated by the lack of calibration across divergent recording environments—constitutes a notable limitation. As noted in the literature, French directive force is often multimodal, relying not only on nuclear pitch reshaping but also on increased subglottal pressure and intensity (Martin, 2018). By focusing exclusively on F0, our analysis may overlook a potential loudness-based compensatory strategy. It is plausible that lower-proficiency learners, struggling to execute the fine-grained pitch modulation required for French commands (Tier 3), may default to a coarser strategy of simply increasing vocal intensity to signal urgency. If such a trade-off exists, our results might underestimate the learners’ pragmatic intent, even if their prosodic implementation remains non-target-like. However, the absence of intensity data does not undermine the central finding regarding the Tone-Intonation interface. The primary cognitive challenge for Mandarin learners is the functional reallocation of pitch from a lexical to a post-lexical domain. The persistence of structural flatness and prosodic merging in the F0 dimension confirms that, regardless of intensity levels, learners face a functional mapping challenge in decoupling pitch from the syllable to encode intonational meaning. Future studies in controlled laboratory settings should incorporate intensity to verify whether this processing constraint restricts all prosodic dimensions globally or whether it specifically targets the high-cost resource of tonal modulation.

Second, the limited number of stimuli, resulting in four items per condition, warrants caution regarding the generalizability of the findings. This constrained corpus was a necessary methodological trade-off to satisfy stringent phonological criteria: to ensure precise F0 extraction, stimuli were constructed with a high density of sonorant segments, severely limiting the range of naturalistic lexical options. Furthermore, given the cognitive demand of the scenario-priming protocol, a larger corpus could have induced fatigue effects, potentially confounding the proficiency-based differences. While our LMMs partially mitigated item-specific idiosyncrasies by including Item as a random intercept, we acknowledge that the current results may still be influenced by specific lexical properties of the selected sentences. Future replication studies utilizing a broader array of lexical items and syntactic structures are necessary to confirm that the observed prosodic merging is a generalized interlanguage rule rather than an artifact of specific sentence types.

Third, to maximize ecological validity, this study utilized naturalistic syntactic structures rather than minimal pairs. While this approach reflects authentic language use, it introduces a syntactic confound (e.g., SVO vs. Imperative mood) that may partially account for native F0 differences. Future investigations could employ controlled minimal pairs (e.g., *Il pleut fort*. vs. *Il pleut fort!*) to isolate the specific contribution of intonation to illocutionary force.

Fourth, the inferential nature of the processing constraints requires empirical validation. While the Complexity-Modality Trade-off observed in our data strongly implies processing overload, we did not employ direct psycholinguistic measures. Subsequent studies utilizing dual-task paradigms or reaction-time metrics are essential to quantify the precise processing threshold at which prosodic precision is sacrificed.

Finally, the MPAH model presented here is a hypothesized trajectory derived from cross-sectional data. The absence of an advanced learner group (C1–C2) limits our capacity to determine whether the observed pragmatic plateau is permanent or permeable. Future longitudinal research tracking the same cohort, as well as comparative studies across diverse L1-L2 combinations, is required to evolve the MPAH from a descriptive framework into a comprehensive predictive model of L2 prosodic competence.

### Pedagogical implications: a tiered instructional framework

Translating the theoretical insights of the MPAH framework into classroom practice, we propose a tiered instructional roadmap designed to systematically dismantle the tone-to-intonation barrier.

#### Prioritizing macro-prosodic scaffolding (Tiers 1 and 2)

Instructional expectations must be calibrated to the learner’s processing constraints. During the initial acquisition of imperative syntax (Tier 1), a premature focus on micro-prosodic precision risks exacerbating cognitive overload. Once syntactic structures are grounded, instruction should shift immediately to macro-prosodic organization (Tier 2). Teachers should encourage a top-down planning strategy to mitigate the L1 syllable-based rhythmic bias. Techniques such as hummed production (de-lexicalized intonation) or rhythmic gestures are essential here: they allow learners to prioritize global phrasal continuity and the establishment of the IP structural framework without the concurrent interference of lexical tones.

#### Targeted pragmatic mapping (Tier 3)

To overcome the developmental hurdle in pragmatic prosody, learners require explicit training in form-function mapping. We recommend the use of contextualized minimal pairs—sentences with identical phonetic strings but distinct pragmatic intents (e.g., *Il pleut fort.* [Observation] vs. *Il pleut fort!* [Exclamation/Warning]). Such high-variability drills force the learner to attend specifically to the expansion of prosodic space and the overall pitch range expansion and fine-grained nuclear modulation required for directive force, thereby facilitating the functional decoupling of pitch from the syllable.

#### Sensorimotor integration via visual feedback

Given the entrenched nature of L1 perceptual filters (Stress Deafness), auditory input alone is often insufficient. Integrating Visual-Acoustic Feedback (VAF) tools (e.g., Praat, real-time pitch trackers) allows learners to visualize the physical contrast between their merged flat templates and the native dynamic contours. This multimodal approach promotes cognitive re-weighting, helping learners consciously override L1 tonal constraints and establish new sensorimotor targets for L2 intonation.

## Data Availability

The raw data supporting the conclusions of this article will be made available by the authors, without undue reservation.

## Appendix. Stimuli

Part 1: Declarative Statements.

Goal: To elicit a gradual pitch declination and a neutral, low-falling boundary tone (L*L%).

(1) C’est. Paul qui a fini le travail.

Scenario: Your boss enters the office and asks who completed the project report. You provide a simple, factual answer: “It was Paul who finished the work.”

(2) Il pleut fort.

Scenario: You are standing by the window looking outside. You casually remark to a friend sitting in the room: “It’s raining hard.”

(3) J’ai beaucoup appris.

Scenario: At the end of a semester, your teacher asks for your feedback on the course. You reflect on your experience and state: “I learned a lot.”

(4) La vue est tellement belle.

Scenario: You reach the top of a hill with a companion. You take a moment to admire the scenery and say quietly: “The view is so beautiful.”

(5) C’est une grande surprise.

Scenario: A colleague tells you that a mutual friend won the lottery. You acknowledge the unexpected news calmly: “It is a big surprise.”

(6) Il ne boit pas de café.

Scenario: At a party, the host offers your friend a cup of coffee. You step in to explain your friend’s dietary habit: “He does not drink coffee.”

(7) Il pleut sans cesse.

Scenario: You are on vacation and it has not stopped raining for three days. You tell your family over the phone in a resigned tone: “It rains without ceasing.”

(8) Il pleut tous les jours.

Scenario: Someone asks you what the weather is like in London during the winter. You provide a general fact based on your experience: “It rains every day.”

Part 2: Directive Commands.

Goal: To elicit increased intensity, sharp pitch falls (L* LL%), and insistence on the final syllable.

(1) Faites attention.

Scenario: You see a pedestrian about to step into a deep puddle or off a high curb. You shout a sharp warning to stop them: “Watch out!”

(2) Laissez-moi tranquille.

Scenario: Someone has been pestering you while you are trying to concentrate on a difficult task. You finally lose your patience and bark: “Leave me alone!”

(3) Lavez-vous les mains avant de manger.

Scenario: Dinner is served, but the children are trying to sit down with dirty hands. As a parent, you give a stern, non-negotiable order: “Wash your hands before eating.”

(4) Ne fume pas!

Scenario: You see someone about to light a cigarette in a gas station or a strictly prohibited area. You intervene immediately: “Do not smoke!”

(5) Sois calme!

Scenario: Your friend is starting to panic or get into a heated argument over a small mistake. You demand that they get their emotions under control: “Be calm!”

(6) Entrez et prenez une chaise.

Scenario: You are a busy manager. A student knocks on your door for an appointment. You give them a direct, authoritative instruction without looking up: “Come in and take a chair.”

(7) Fermez la porte.

Scenario: There is a loud noise coming from the hallway that is interrupting your meeting. You order the person nearest the door to shut it immediately: “Close the door.”

(8) Ne vous plaignez pas.

Scenario: A friend keeps grumbling about a minor inconvenience (e.g., the weather), which becomes annoying. You lose patience and order them to stop the negativity: “Do not complain.”
